# KAN–EEG: towards replacing backbone–MLP for an effective seizure detection system

**DOI:** 10.1098/rsos.240999

**Published:** 2025-03-12

**Authors:** Luis Fernando Herbozo Contreras, Jiashuo Cui, Leping Yu, Zhaojing Huang, Armin Nikpour, Omid Kavehei

**Affiliations:** ^1^ School of Biomedical Engineering, Faculty of Engineering, The University of Sydney, Sydney, NSW 2006, Australia; ^2^ Comprehensive Epilepsy Service and Department of Neurology, Royal Prince Alfred Hospital, Sydney, NSW 2050, Australia; ^3^ Faculty of Medicine and Health, Central Clinical School, The University of Sydney, Sydney, NSW 2006, Australia; ^4^ The University of Sydney Nano Institute, Sydney, NSW 2006, Australia

**Keywords:** Kolmogorov–Arnold networks, robustness, flexibility, seizure, out-of-distribution

## Abstract

The landscape of artificial intelligence (AI) research is witnessing a transformative shift with the emergence of the Kolmogorov–Arnold network (KAN), presenting a novel architectural paradigm aimed to redefine the structural foundations of AI models, which are based on multilayer perceptron (MLP). Through rigorous experimentation and evaluation, we introduce the KAN–electroencephalogram (EEG) model, a tailored design for efficient seizure detection. Our proposed network is tested and successfully generalized on three different datasets, one from the USA, one from Europe, and one from Oceania, recorded with different front-end hardware. All datasets are scalp EEG in adults and are from patients living with epilepsy. Our empirical findings reveal that while both architectures demonstrate commendable performance in seizure detection, the KAN model exhibits high-level out-of-sample generalization across datasets from diverse geographical regions, underscoring its inherent efficacy and adaptability at the backbone level. Furthermore, we demonstrate the resilience of the KAN architecture to model size reduction and shallow network configurations, highlighting its versatility and efficiency by preventing over-fitting in-sample datasets. This study advances our understanding of innovative neural network architectures and underscores the pioneering potential of KANs in critical domains such as medical diagnostics.

## 1. Introduction

Epilepsy is a neurological disorder causing recurring seizures, affecting millions globally. In about 30–35% of cases, standard anti-epileptic drugs (AEDs) fail to control abnormal brain activities, resulting in drug-resistant epilepsy. Despite advancements in AED development and testing, improvements in their effectiveness have been limited [1]. Unpredictable and unprovoked seizures significantly impact patients’ quality of life, employment and overall well-being, posing risks such as falls and sudden unexpected death in epilepsy [2,3]. An accurate system for detecting and counting seizures can greatly enhance decision-making, treatment planning and disease management, leading to better patient outcomes. Electroencephalogram (EEG) signals are commonly used by scientists to diagnose neurological diseases such as seizures. It is a technique that records brain electrical activity by placing electrodes on the scalp to capture electrical signals generated by neuronal activity [4]. These electrical signals reflect the brain’s functional state and information processing, making EEG an important tool for studying brain function [5]. Variations in EEG signals in time and frequency can reveal brain activity patterns associated with sleep, cognition, emotion and pathological states such as epilepsy [6]. Artificial intelligence (AI) has undeniably made significant strides in healthcare, becoming a new era of innovation and patient care. Its impact on the healthcare industry has been overwhelmingly positive, with numerous benefits spanning various aspects of medical practice, research and patient outcomes [7–9]. Among the various AI techniques, machine learning models, particularly neural networks, have shown great promise in analysing and interpreting complex medical data. Multilayer perceptron (MLP) networks are the backbones of today’s AI architectures [10,11] and have been extensively used for their effectiveness in detecting and classifying abnormalities from biosignals. The MLP, a feed-forward artificial neural network, consists of multiple layers of neurons that process input data through weighted connections and activation functions. It excels in capturing the relationships within the data, making it well-suited for analysing the intricate patterns present in medical signals. Despite its advantages, the MLP has limitations, particularly in model interpretability and efficiency. To address these challenges, a new model known as the Kolmogorov–Arnold network (KAN) has been proposed as a promising alternative to MLPs [12]. Unlike MLPs, which use fixed activation functions on the hidden layers, KANs employ learnable activation functions, replacing linear weights with univariate functions parametrized as splines. This architectural difference allows them to achieve greater accuracy and interoperability.

**Figure 1.**
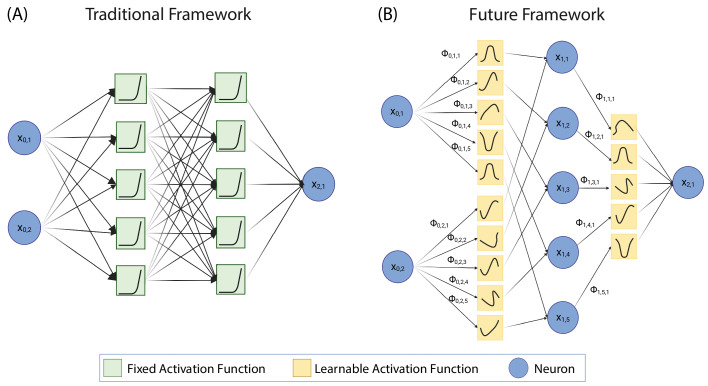
Representation of the past and future in neuro-AI. The multilayer perceptron was built based on non-learnable activation functions and relies on deep, fully connected networks for accurate performance (A). On the contrary, KAN emerges as a potential solution for more explainable, shallow, and efficient architectures at the core level (B).

KANs have demonstrated the ability to outperform MLPs with smaller network sizes, making them more computationally efficient [12]. Despite its advantages, the MLP has limitations, particularly in model interpretability and efficiency. Furthermore, it has demonstrated the ability to outperform MLPs with smaller network sizes, making them more computationally efficient [12]. In the context of EEG signal analysis, KANs offer potential advantages over MLPs. Their learnable activation functions and efficient representation of data structures enable better handling of the complex, high-dimensional data characteristic of EEG signals. This can improve detection accuracy and more insightful interpretations of the underlying neural activity. The subsequent sections will delve deeper into the comparative performance of our proposed architectures and MLPs in detecting epileptic seizures, highlighting the strengths and potential of this critical application. The diagram in figure 1 delineates the structural differences between the KAN and the MLP architectures. The KAN structure demonstrates learnable activation functions, which can constitute interpretable systems and face MLP architecture challenges through enhanced capabilities for continual learning and efficiency at shallow, sparse connections.

### 1.1. Background

Epileptic seizure detection has seen substantial progress with the rise of machine learning models, particularly those using EEG signals. EEG tests record the electrical activity produced by neurons, typically non-invasively, by placing multiple electrodes on the scalp [4]. EEGs play a crucial role in detecting epileptic foci and categorizing epilepsy types, such as focal, generalized and unknown seizures [13–15]. These recordings are essential for diagnosing epilepsy, monitoring ongoing conditions, making predictions and effectively responsive neurostimulation. Recent advancements in deep learning have introduced various architectures to improve EEG-based seizure detection. Convolutional neural networks (CNNs) are prominently used, transforming EEG signals into different dimensional forms for detailed analysis [16–21]. Recurrent neural networks (RNNs), including their advanced versions like long short-term memory (LSTM) and gated recurrent units, are adept at capturing temporal dependencies in EEG data, making them suitable for sequential data analysis [22–25]. Unsupervised learning methods, such as deep belief networks [26,27] and auto-encoders (AEs) [28–30], are employed to extract and reconstruct features from raw signals.

Table 1 summarizes various traditional models applied to different bio-signal applications, pre-processing methods, datasets and indicators. In addition, hybrid models that combine CNNs with RNNs or AEs use the spatial feature extraction capabilities of CNNs and the temporal modelling strengths of RNNs or the reconstruction abilities of AEs [31,32,38–41]. For example, convolutional long short-term memory (ConvLSTM) networks integrate the spatial feature extraction power of CNNs with the temporal sequence modelling capabilities of LSTMs [32]. This integration has enhanced the model’s performance in detecting seizures.

**Table 1.** Different types of neural networks applied to EEG signals for epileptic seizure detection.

model	application	pre-processing ^a^	dataset ^b^	metrics ^c^	limitations
3D-CNN+RNN [31]	seizure detection	STFT 2D mapping	CHB-MIT SNUH	ACC: 99.4% ACC: 99.2%	small cohort
ConvLSTM [32]	seizure detection	ICA, STFT	TUH EPILEPSIAE RPAH	AUC: 84% AUC: 81% AUC: 82%	post-processing dependency
SNN-ConvLSTM [33]	seizure detection	STFT	FB CHB-MIT EPILEPSIAE	AUC: 92.7% AUC: 89% AUC: 81.1%	low accuracy
TSD [34]	seizure detection	STFT	TUH	AUC: 92.1%	no out-of-sample test
ResNet-LSTM [35]	seizure detection	ICA, STFT	U-Bonn	ACC: 97%	memory usage
GraphS4mer [36]	seizure detection sleep staging ECG classification	STFT	TUH DOD-H ICBEB	AUC: 90.6% macro-F1: 81.8% AUC: 97.7%	memory increases quadratically
SVM [37]	seizure detection	DWT	CHB-MIT	ACC: 98%	small cohort

^a^ Pre-processing methods: short-time Fourier transform (STFT), independent component analysis (ICA), discrete wavelet transform (DWT).

^b^ Datasets: Temple University Hospital (TUH), Children’s Hospital Boston (CHB-MIT), Royal Prince Alfred Hospital (RPAH), Seoul National University Hospital (SNUH), University of Bonn (U-Bonn), Freiburg (FB), Dreem Open Dataset-Healthy (DOD-H), International Conference on Biomedical Engineering and Biotechnology (ICBEB).

^c^ Metrics: accuracy (ACC), area under the curve (AUC), macro-averaged F1-score (Macro-F1).

Transformer-based networks, a more recent innovation, incorporate attention mechanisms to better capture complex patterns in EEG data, further improving detection accuracy [34,42–44]. Despite these significant advancements, traditional seizure detection models still encounter challenges in generalization and real-time implementation. Models trained on large datasets often struggle with low area under the receiver operating curve (AUROC) and high false-positive rates, which limits their clinical applicability. Thus, there is a pressing need for models that can balance sensitivity and specificity and be broadly applicable across diverse patient populations and real-world scenarios [32,45].

Although the traditional MLP-based model has significantly contributed to the development of EEG-based seizure detection systems, the emergence of KAN and other advanced models provides a promising direction for improving the accuracy, efficiency, and applicability of epilepsy detection technology. The following section will provide a detailed comparison between MLP and KAN, emphasizing the advantages and potential of KAN in EEG signal analysis of epilepsy detection.

### 1.2. Limitations of the previous studies

Previous models used in machine learning-based systems, particularly those relying on MLP architectures, have been constrained by their reliance on fixed activation functions. These functions, such as rectified linear unit (ReLU) or sigmoid, are pre-defined and static, limiting the model’s flexibility and adaptability. Furthermore, traditional MLP architectures lack the capability for continuous learning, meaning that once a model is trained, it cannot adapt based on new incoming data without retraining. This limitation poses a significant challenge for real-time applications or scenarios where the data distribution changes over time. Without the ability to continuously learn and adapt, the models can become less accurate, leading to performance degradation. Therefore, there is growing interest in developing architectures that dynamically adjust their activation functions and support continuous learning, enabling more flexible, robust and efficient models for complex, real-world applications.

### 1.3. Novelty and significance

In this study, our primary aim is to introduce a novel backbone architecture for advancing the field of seizure detection, focusing on addressing challenges related to generalization and real-time implementation. To the best of our knowledge, this is the first study that uses a novel non-backbone MLP for healthcare applications and the first study to be used in seizure detection. We propose the utilization of KANs as a key component for identifying epileptic seizures from pre-recorded EEG signals. We incorporate the short-time Fourier transform (STFT) technique to process the EEG data effectively. Rather than solely emphasizing the challenges in generalization and implementation, we highlight the innovative potential of efficient-shallow architectures adaptable as a foundational framework for future AI models in seizure detection. Through our experimentation with the three different continental datasets, we aim to showcase the inherent efficacy of KANs and their capacity to serve as an efficient architecture for developing advanced seizure detection systems. We found the following advantages for our proposed method: (*higher accuracy*) preliminary findings suggest that our model demonstrates comparable AUROC values to traditional MLP models, indicating a similar level of performance in seizure detection. This comparative analysis suggests that while KANs do not necessarily outperform MLP models, they offer a comparable level of reliability, thereby contributing to the advancement of seizure detection methodologies; (*efficiency*) our proposed architecture requires a smaller network size, thereby making them computationally efficient and suitable for real-time applications; (*less training, high performance*) our proposed architecture achieves high accuracy while requiring only a small portion of the training dataset, outperforming models that typically require larger datasets for similar results; (*out-of-sample seizure detection*) generalization beyond the training dataset was assessed by training our model on the USA dataset and evaluating its performance on independent datasets from Europe and Oceania, using the same trained weights. The outcomes indicate encouraging support for this test.

## 2. Methods

### 2.1. Datasets

**Figure 2.**
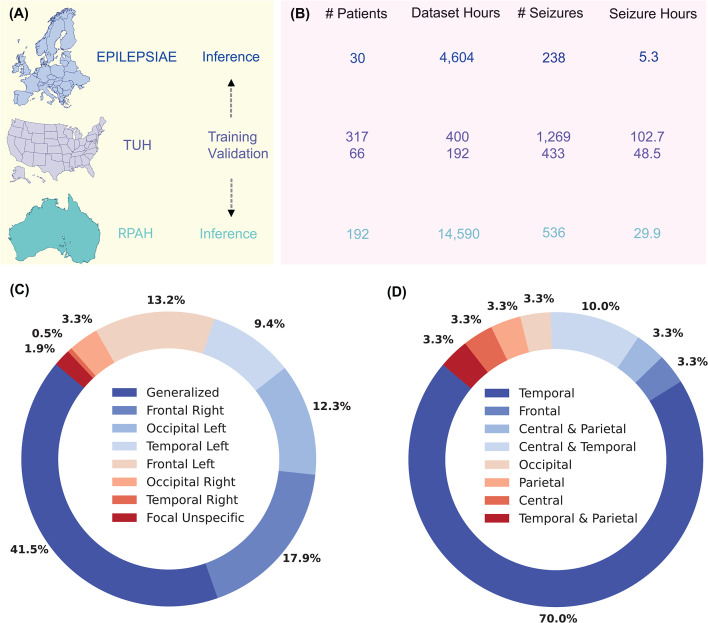
Summary of the datasets being used. The TUH dataset was set as the training and validation dataset and subsequently used for inference in both RPAH and EPILEPSIAE datasets (A). Testing across all datasets incorporates extensive background and seizure session data, facilitating comprehensive analysis of model efficacy in diverse clinical settings (B). Types of sinsights into the number ofeizures across the RPAH dataset (C) and EPILEPSIAE dataset (D).

Three datasets were used in this work: the Temple University Hospital (TUH) EEG Corpus, the scalp-EPILEPSIAE dataset, and the Royal Prince Alfred Hospital (RPAH) dataset. Figure 2 summarizes the TUH dataset, detailing key statistics. This dataset is divided into training and validation sets, offering comprehensive insights into the number of hours used for both patients with seizures and non-seizures and the number of patients that present seizures and non-seizures. The TUH dataset is the primary training dataset, providing a diverse range of seizure signals for various seizure types. With its large volume of files, the TUH dataset from the USA offers extensive data for robust model training. We used 400 h of EEG data for our training process, comprising 120 000 samples with a 12 s window. This dataset included approximately 75% background activity and 25% seizure activity. Notably, this represents a significantly smaller data volume than other models. Despite the reduced dataset size, we successfully validated the KAN–EEG model’s efficiency in seizure detection with reduced training data. The validation was conducted using 192 hours of EEG data, corresponding to 57 306 samples with a 12 s window, and maintained the same 75–25% ratio of background to seizure information.

The EPILEPSIAE and RPAH datasets, consisting of adult EEG, are used for inference tests. Both datasets share common characteristics, such as using identical montages and adult patient data, ensuring consistency and comparability in analysis. Integrating these complementary datasets enhances the comprehensiveness and reliability of the AI model for seizure detection. The EPILEPSIAE dataset consists of 30 patients, where 19 and 11 are male and female, respectively. The RPAH dataset from one of Australia’s major hospitals reliably maintained one of the largest datasets from adult epilepsy patients nationwide. This work uses nine years (2011−2019) of data, testing nearly 14 590 h of EEG data from 192 patients over 1006 sessions, each averaging around 15 h of recording. Out of 212 patients, 20 were excluded for reasons including excessive seizures (more than 11 seizures per 24 h), missing electrode data, or seizures confirmed only by video.

The Australian dataset is about 16 times larger than the US training dataset, with longer interictal periods and background data, making evaluating false positives highly robust. The distribution of the RPAH dataset across three domains—seizure type and frequency, age, gender and seizure occurrence within a 24 h cycle—highlights essential patterns. Notably, seizure occurrences are derived from intermittent monitoring, providing insights into the likely timing of seizures. Ethical approval was obtained to access this clinical data.

### 2.2. The Kolmogorov-Arnold network-electroence phalogram structure

#### 2.2.1. Characteristics of Kolmogorov-Arnold networks

KANs are founded on the Kolmogorov–Arnold representation theorem, which posits that any multivariate continuous function can be broken down into a finite composition of continuous univariate functions and addition operations [12]. This foundational principle allows it to substitute traditional linear weights with spline-parametrized univariate functions. Moreover, it employs adaptive univariate activation functions along the network edges, enhancing flexibility and precision. These functions adjust based on the data, leading to more accurate approximations. Spline functions enable dynamic adaptation to the data, providing refined representations that effectively capture smooth transitions. Additionally, KANs require fewer parameters than MLPs, improving computational efficiency and model interpretability. The learnable functions can also be visualized for better understanding. The operation of a KAN layer can be described by equation (2.1):


(2.1) $${\hat{x}}_{i}=\sum_{j=1}^{n}\varphi_{ij}(x_{j}),$$

where ${\hat{x}}_{i}$ represents the activation value at node i, and $\varphi_{ij}$ denotes the learnable activation function on the edge connecting node j to node i.

#### 2.2.2. Mathematical formulation of Kolmogorov-Arnold networks

KANs use the Kolmogorov–Arnold representation theorem to break down a high-dimensional function into a sum of univariate functions. This decomposition is given by equation (2.2):


(2.2) $$f(x)=\sum_{q=1}^{2n+1}\Phi_{q}\left(\sum_{p=1}^{n}\varphi_{q,p}(x_{p})\right).$$

In this formulation, $\Phi_{q}$ and $\varphi_{q,p}$ are univariate functions parameterized as splines, while $x_{p}$ represents the input features. The inner functions $\varphi_{q,p}(x_{p})$ transform the input features into intermediate representations, which are then aggregated and processed by the outer functions $\Phi_{q}$. This structured approach enables KANs to effectively capture compositional structures and univariate functions, providing a robust framework for function approximation [12]. An overall representation of the KAN structure is seen in figure 1.

### 2.3. Pre-processing

**Figure 3.**
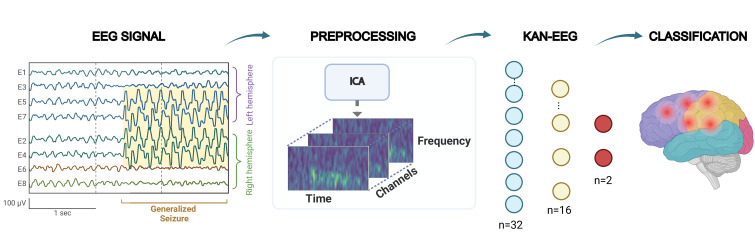
KAN–EEG seizure system. We analysed different kinds of seizures using the TUH dataset for training. We then preprocessed our model using ICA and STFT. Subsequently, we incorporate a shallow KAN algorithm that leads to efficient results.

We used two signal processing techniques, independent component analysis (ICA) and STFT, to address the challenges associated with raw EEG data. Initially, the EEG signals were divided into 12 s segments, and the ICA algorithm was applied to decompose the signals into 19 independent components using blind source separation. ICA separates EEG signals into statistically independent components, as represented in equation (2.3):


(2.3) $$\begin{array}{lcr} & T\approx MA^{T}\;, & \end{array}$$

where T contains the EEG data, M contains the time information, and A contains the weights for topographic maps. Pearson correlation was used to identify independent sources strongly associated with eye movement, detected from the ‘FP1’ and ‘FP2’ EEG channels. These sources related to eye movement were removed, resulting in EEG signals free from such artefacts. Subsequently, the STFT was applied to the cleaned EEG signals. This involved using a window length of 250 samples (equivalent to 1 s) with a 50% overlap and eliminating the DC component of the transform. As a result, the data dimensions were (N×23×125), where N represents the number of electrodes, 23 represents the time index, and 125 represents the frequencies. Data pre-processing is performed separately from the KAN model, ensuring the data is adequately prepared before input into the KAN model for further training/inference, as depicted in figure 3.

## 3. Experiments and results

### 3.1. Training and validation in sample

**Figure 4.**
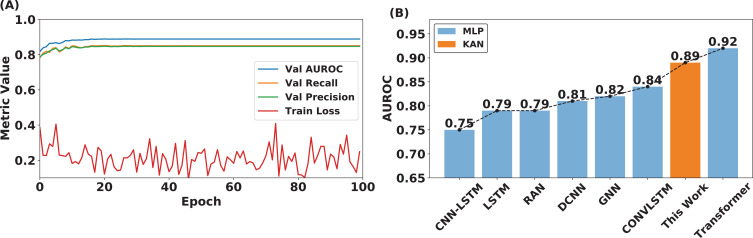
In-sample results on the TUH dataset. Our model was trained on 400 h of data, significantly less than the data used to train other models, such as ConvLSTM and transformer, with 752 and 910, respectively (A). A comparison of a KAN–EEG (Architecture **I−764−256-O** (table 2) training with other MLP-based models [32,34,46–48] demonstrates better performance with less training data.

Our model was trained and validated using the TUH dataset. We achieved an impressive AUROC score of 0.89 in figure 4B. It is important to highlight that our model was trained on 400 h of data, considerably fewer than the 752 h used for the ConvLSTM model and the 910 h for the transformer model. These preliminary results highlight the exceptional performance of our approach, which not only rivals but also exceeds that of contemporary methods by considering a smaller training dataset but a comparable and even greater test dataset. Our model demonstrates superior efficacy in accurately detecting epileptic seizures, underscoring its potential for clinical applications. The structure used to obtain the outlined results is highlighted in table 2, and we use this as our baseline for our following assessment.

**Table 2.** Model assessment robustness in-sample validation using the TUH dataset by decreasing layers and neurons leads to efficiency. (I: input data. We used 23 time and 125 frequency bins, followed by 19 EEG channels; O: output neurons. We used two output neuronsto represent states of seizure and non-seizure);

model structure	layers	hidden neurons	neuron reduction	model size reduction	AUROC	AUPRC	precision	recall	F1-score
I−764−256-O^a^ (large)	2	1020	baseline	baseline	88.9	77.4	85.0	84.7	85.1
I−32−32-O^b^	2	64	6.27 %	4.5 %	86.9	73.5	83.2	83.0	83.3
I−32−16-O^c^ (small)	2	48	4.71 %	4.38 %	86.7	73.1	82.9	83.1	82.8
I−256-O	1	256	25 %	29.8 %	85.3	70.1	81.3	81.2	80.9

^a^ Out-of-sample generalizations are shown in tables 3 and 4.

^b^ Out-of-sample generalizations are shown in tables 3 and 4.

^c^ Out-of-sample generalizations are shown in tables 3 and 4.

**Table 3.** Out-of-sample generalization results (AUROC) on the RPAH dataset.

year	Liquid-Time Constant and Forward Propagation Through Time (LTC-FPTT) [49]	conv-LSTM [32]	KAN–EEG^a^	KAN–EEG^b^	KAN–EEG^c^
2011	0.88	0.90	0.75	0.95	0.93
2012	0.92	0.91	0.64	0.90	0.88
2013	0.92	0.89	0.62	0.89	0.88
2014	0.78	0.73	0.61	0.80	0.78
2015	0.79	0.82	0.56	0.91	0.89
2016	0.81	0.85	0.55	0.86	0.84
2017	0.80	0.68	0.59	0.72	0.71
2018	0.80	0.72	0.56	0.80	0.79
2019	0.80	0.78	0.62	0.81	0.79
overall	0.83	0.82	0.60	0.85	0.83

^a^ Architecture I-764-256-O (table 2): two layers (764 and 256 neurons).

^b^ Architecture I-32-32-O (table 2): two layers (32 and 32 neurons).

^c^ Architecture I-32-16-O (table 2): two layers (32 and 16 neurons).

**Table 4.** Out-of-sample generalization results (AUROC) on the EPILEPSIAE dataset.

pat-ID	LTC-FPTT [49]	conv-LSTM [32]	KAN–EEG^a^	KAN–EEG^b^	KAN–EEG^c^
1	0.88	0.88	0.63	0.84	0.79
2	0.82	0.88	0.49	0.58	0.55
3	0.84	0.85	0.60	0.85	0.77
4	0.82	0.89	0.31	0.71	0.58
5	0.79	0.72	0.58	0.73	0.72
6	0.78	0.69	0.42	0.64	0.60
7	0.78	0.81	0.53	0.75	0.76
8	0.79	0.81	0.63	0.88	0.82
9	0.81	0.92	0.65	0.90	0.88
10	0.81	0.68	0.43	0.57	0.49
11	0.81	0.81	0.82	0.96	0.95
12	0.81	0.74	0.48	0.75	0.59
13	0.83	0.86	0.33	0.78	0.63
14	0.83	0.85	0.65	0.84	0.82
15	0.83	0.71	0.58	0.81	0.82
16	0.83	0.85	0.77	0.93	0.91
17	0.83	0.83	0.44	0.65	0.63
18	0.83	0.82	0.52	0.82	0.68
19	0.84	0.91	0.74	0.88	0.87
20	0.84	0.68	0.34	0.71	0.65
21	0.84	0.83	0.47	0.83	0.71
22	0.84	0.95	0.62	0.74	0.70
23	0.85	0.84	0.60	0.89	0.84
24	0.84	0.83	0.76	0.85	0.82
25	0.84	0.62	0.56	0.75	0.73
26	0.84	0.84	0.38	0.67	0.57
27	0.84	0.86	0.60	0.71	0.70
28	0.84	0.91	0.70	0.88	0.88
29	0.84	0.72	0.51	0.79	0.76
30	0.85	0.88	0.43	0.75	0.62
overall	0.83	0.81	0.55	0.78	0.73

^a^ ArchitectureI-764-256-O (table 2): two layers (764 and 256 neurons).

^b^ ArchitectureI-32-32-O (table 2): two layers (32 and 32 neurons).

^c^ ArchitectureI-32-16-O (table 2): two layers (32 and 16 neurons).

The training processing was performed for 100 epochs, where in figure 4A can be seen the trend across different metrics. Among these, the discernible reduction in the training loss is evident, gradually converging towards optimal thresholds. Noteworthy is the observation that metrics such as recall, precision, and AUROC manifest a discernible tendency to reach the convergence phase approximately around the 20th epoch. Given the nature of a small architecture, training losses have an average convergence between 0.2 and 0.3.

### 3.2. Robustness

The findings presented in table 2 offer compelling insights into the robustness of the KAN–EEG architecture in seizure detection. By systematically reducing the complexity of the model through decreases in both layers and hidden neurons, we sought to evaluate the architecture’s adaptability and efficiency under varying structural configurations. Our analysis reveals that despite the reduction in model complexity, the KAN architecture consistently maintains a high level of performance across key evaluation metrics, as reflected by the consistently high AUROC values across all models. For instance, the largest tested model has an AUROC of 0.89, while the smallest model tested is 0.85. This indicates the model’s ability to differentiate between seizure and non-seizure states effectively, even when the architectural complexity is significantly reduced.

Moreover, while marginal decreases are observed in metrics such as precision and recall with model simplification, the overall performance, as quantified by the F1-Score, remains robust. This suggests that our architecture not only preserves its ability to detect seizures accurately but also maintains a balanced trade-off between precision and recall. It is crucial for real-world applications where false positives and negatives have significant implications. These results underscore the resilience of the proposed model to structural modifications, highlighting its adaptability and efficiency in resource-constrained environments. The ability of the model to maintain high-performance levels even with reduced computational complexity holds promising implications for practical deployment.

### 3.3. Compact networks leads to out-of-sample continental generalization

*Can the KAN–EEG generalize beyond in-sample testing?* Out-of-sample generalization is crucial in seizure detection models, as it assesses the model’s ability to perform effectively on unseen data that was not included during training. This is essential for establishing more robustness and reliability of the model in real-world clinical settings, where the variability of patient populations, seizure types and environmental factors can significantly impact performance. To evaluate generalization performance, we tested our initial model with two layers (764 and 256 neurons, respectively) on two distinct out-of-sample datasets. The RPAH dataset results are presented in table 3, and the EPILEPSIAE dataset results are shown in table 4. The results shown demonstrate some out-of-sample testing ability, albeit not efficiently, in RPAH and EPILEPSIAE datasets with AUROC results of 0.60 and 0.55, respectively. The observed results primarily stem from an architectural design showing indications of overfitting on the training dataset. This assertion suggests that the model has become overly specialized in capturing specific information inherent in the training/validation data, consequently compromising its ability to generalize in different datasets. As a result, addressing overfitting is crucial for enhancing the model’s robustness and applicability in real-world scenarios, with unseen-world datasets with varying patterns of seizure that can be specific to each patient.

To address the challenges of inefficient generalization, we modified the model to have two layers with 32 and 16 neurons, respectively. Additionally, we provided an extra evaluation to confirm that our findings are assertive by testing in a model with two layers, each with 32 neurons. As these architectures showed robustness in in-sample testing, with an AUROC of 0.87, we applied them to the generalization test using weights from training when the AUROC metric was not increasing.

As discussed previously, the KAN–EEG model with two layers (764 and 256 neurons) had a lower AUROC than the LTC-FPTT and ConvLSTM models. Interestingly, the KAN–EEG models with two layers (32 and 16 neurons, and 32 and 32 neurons) demonstrated comparable or superior AUROC values relative to these models. For the RPAH dataset, we demonstrated that the compact proposed networks have an AUROC of 0.85 and 0.83, respectively, outperforming existing models. For the EPILEPSIAE dataset, although we did not surpass previous studies, our results were closer to 0.78 and 0.75, respectively.

This suggests that overfitting may occur in models with more complex neuron structures, adversely affecting their generalization performance. These findings are significant as they indicate the potential for future deployable on-edge KAN algorithms, where memory size compression and robustness are crucial. These findings suggest the potential for replacing MLP-based models for seizure detection with the KAN–EEG algorithm. Our framework remains robust and demonstrates promise for practical implementation in real-world applications. It is essential to highlight that our data was not trained on the full TUH dataset, indicating that there is even further room for improvement in achieving greater results.

### 3.4. Computation time in training

In this study, we used a Tesla P100 GPU with 16 GiB of memory to compare the training performance of two neural network configurations of the KAN–EEG model: a larger network with 764−256 neurons and a smaller network with 32−16 neurons. Using a batch size of 64, the training time per epoch for the larger network averaged 4 min and 12 s, while the smaller network completed an epoch in 1 min and 1 s. Regarding GPU memory consumption, the more extensive network required approximately 8.8 GiB, whereas the smaller network used only 1.6 GiB. These results underscore the efficiency of this neural network architecture, which is crucial for real-time applications. By contrast, spiking neural networks often require significantly more training time, especially when analysing extended sequences with many timesteps. The fast training performance of the KAN–EEG model makes it well-suited for real-time scenarios.

## 4. Discussion

In this study, we pioneer the application of shallow KANs to EEG data, using this model’s unique structure and capabilities inspired by the Kolmogorov–Arnold representation theorem. The proposed architecture not only achieves a higher accuracy in-sample dataset but can also be generalized across different scalp-EEG, as smaller KANs can outperform much larger MLPs in terms of data fitting and partial differential equation solving, owing to faster neural scaling laws [12]. By applying KANs to EEG data, we expect to unlock new potentials in seizure prediction models, given its ability to learn compositional structures and optimize univariate functions [12]. This novel application could provide more accurate and interpretable predictions, advancing the neural network-based seizure prediction field. The model’s resilience against catastrophic forgetting will also ensure stable and continuous learning, a critical requirement for medical applications involving long-term EEG monitoring. To this extent, our next steps will include deploying this architecture on memristor devices. Recent studies have demonstrated the potential of incorporating a KAN network with neuromorphic hardware. Given that memristors are a key component in developing neuromorphic platforms, this approach introduces exciting opportunities for the deployment of such models on non-conventional hardware [50].

### 4.1. Study and model limitations

This research serves as a proof of concept to evaluate whether the KAN model, which uses a learnable activation function, can form a viable AI-based approach for seizure detection. However, further exploration into the optimal architectural design for improved performance is still needed, although we have demonstrated that shallow networks tend to have better generalization and robustness as described in table 2. This study is limited to seizure detection, but we will incorporate this study’s insights from the architectures for seizure prediction. Deployability has not been considered within this study. However, based on the architecture model, its aim can be tested in future studies. One noticeable drawback we found in our study was a linear increase in memory consumption based on the input data. Therefore, future studies should focus on aiming these challenges.

The data used in this model are in the frequency domain, which used a pre-processing step to transform the raw signals into a format suitable for analysis. This additional pre-processing imposes limitations, especially when considering real-time applications as it can introduce latency and increase computational demands, making the model less practical for real-world scenarios where rapid or continuous data processing is required. Consequently, this limitation affects the model’s usability in time-sensitive or resource-constrained environments, such as portable or embedded systems.

## 5. Conclusion

This study has introduced the KANs as a novel approach for epileptic seizure detection using EEG signals. By using its unique architecture and learning properties, which feature learnable activation functions on edges instead of fixed ones on nodes, we have demonstrated the potential for achieving comparable accuracy and higher accuracy in both in-sample and out-of-sample seizure detection compared to traditional MLP models. Our results indicate that KANs should be a solution for replacing MLPs-based models for seizure detection applications, requiring a smaller network size while maintaining robust performance. The significant advancements presented in this work highlight the potential of KANs to improve clinical outcomes for patients with epilepsy by providing a more accurate, efficient and interpretable tool for seizure detection that enhances decision-making, treatment planning and overall disease management.

## Data Availability

Data and relevant code for this research work are stored in GitHub: [51] and have been archived within the Zenodo repository: [52]. The TUH dataset is publicly available at https://isip.piconepress.com/projects/tuh_eeg/. The EPILEPSIAE dataset is available at cost via https://www.epilepsy-database.eu/. The Department of Neurology at the Royal Prince Alfred Hospital (RPAH) dataset was used under Ethics Review Board approval and is not publicly available. If you have any questions regarding access to the code employed in this research paper, please direct your inquiries to the corresponding author. It is important to note that the author may outline specific terms, conditions or usage restrictions for the code, which will be provided to you as needed.
